# *QuickStats:* Breast Cancer Death Rates* Among Women Aged 50–74 Years, by Race/Ethnicity — National Vital Statistics System, United States, 2006 and 2016

**DOI:** 10.15585/mmwr.mm6721a8

**Published:** 2018-06-01

**Figure:**
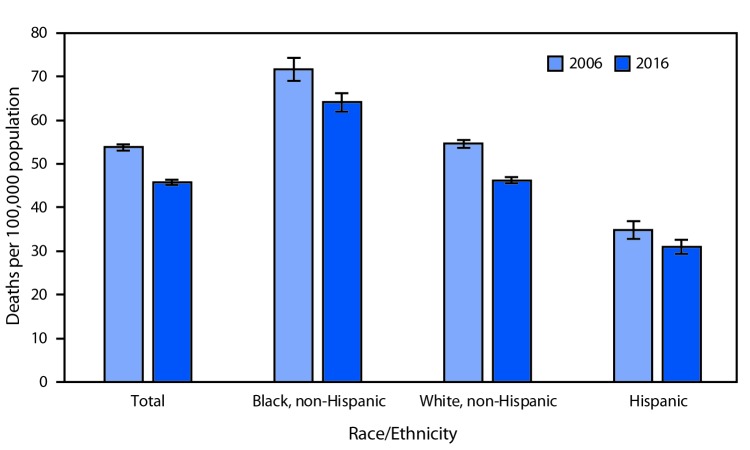
The death rate from breast cancer among all women aged 50–74 years decreased 15.1%, from 53.8 per 100,000 in 2006 to 45.7 in 2016. In both 2006 and 2016, the death rate was higher among non-Hispanic black women compared with non-Hispanic white women and Hispanic women. From 2006 to 2016, the death rate from breast cancer decreased for non-Hispanic white women from 54.6 per 100,000 to 46.2, for Hispanic women from 34.8 to 31.0, and for non-Hispanic black women from 71.7 to 64.1.

**Source:** National Vital Statistics System, 2006 and 2016. https://wonder.cdc.gov/ucd-icd10.html.

